# 2,6-Bis(3-phenyl-1*H*-pyrazol-5-yl)pyridine monohydrate

**DOI:** 10.1107/S1600536808008520

**Published:** 2008-04-10

**Authors:** Jian-Yu Dong, Tian-Pa You

**Affiliations:** a Department of Chemistry, University of Science and Technology of China, Hefei, Anhui 230026, People’s Republic of China

## Abstract

In the title compound, C_23_H_17_N_5_·H_2_O, the pyrazole rings are slightly twisted from the central pyridine ring, forming dihedral angles of 5.3 (2) and 3.5 (2)°. The pyrazole and phenyl rings on each side of the pyridine ring are also approximately coplanar, making dihedral angles of 6.0 (2) and 4.5 (2)°. In the crystal structure, 2,6-bis­(3-phenyl-1*H*-pyrazol-5-yl)pyridine and water mol­ecules are linked together *via* N—H⋯O and O—H⋯N hydrogen bonds, forming a column running parallel to the *a* axis.

## Related literature

For general background, see: Dias & Gamage (2007 ▶); Zhou & Chen (2007*a* ▶,*b* ▶); Zhang *et al.* (2007 ▶).
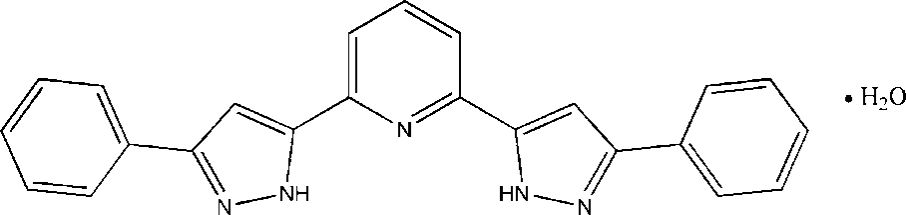


## Experimental

### 

#### Crystal data

C_23_H_17_N_5_·H_2_O
*M* *_r_* = 381.43
Monoclinic, 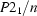

*a* = 8.0581 (17) Å
*b* = 19.975 (2) Å
*c* = 12.451 (2) Å
β = 98.273 (3)°
*V* = 1983.2 (6) Å^3^
*Z* = 4
Mo *K*α radiation
μ = 0.08 mm^−1^
*T* = 298 (2) K
0.42 × 0.11 × 0.07 mm

#### Data collection

Bruker SMART CCD area-detector diffractometer
Absorption correction: multi-scan (**SADABS**; Sheldrick, 2002 ▶) *T*_min_ = 0.966, *T*_max_ = 0.994
9869 measured reflections
3513 independent reflections
1796 reflections with *I* > 2σ(*I*)
*R*_int_ = 0.077

#### Refinement

*R*[*F* ^2^ > 2σ(*F* ^2^)] = 0.063
*wR*(*F* ^2^) = 0.159
*S* = 1.05
3513 reflections
271 parameters
3 restraints
H atoms treated by a mixture of independent and constrained refinement
Δρ_max_ = 0.18 e Å^−3^
Δρ_min_ = −0.18 e Å^−3^

### 

Data collection: *SMART* (Bruker, 2001 ▶); cell refinement: *SAINT* (Bruker, 2001 ▶); data reduction: *SAINT*; program(s) used to solve structure: *SHELXTL* (Sheldrick, 2008 ▶); program(s) used to refine structure: *SHELXTL*; molecular graphics: *SHELXTL* software used to prepare material for publication: *SHELXTL* and *publCIF* (Westrip, 2008 ▶).

## Supplementary Material

Crystal structure: contains datablocks global, I. DOI: 10.1107/S1600536808008520/is2276sup1.cif

Structure factors: contains datablocks I. DOI: 10.1107/S1600536808008520/is2276Isup2.hkl

Additional supplementary materials:  crystallographic information; 3D view; checkCIF report

## Figures and Tables

**Table 1:** Hydrogen-bond geometry (Å, °)

*D*—H⋯*A*	*D*—H	H⋯*A*	*D*⋯*A*	*D*—H⋯*A*
O1—H22⋯N5^i^	0.84 (3)	2.13 (3)	2.878 (4)	148 (4)
O1—H23⋯N3^ii^	0.84 (4)	2.20 (2)	2.956 (4)	150 (4)
N2—H17⋯O1	0.86	2.07	2.918 (4)	167
N4—H5⋯O1	0.86	2.16	3.007 (4)	170

Symmetry codes: (i) 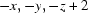; (ii) 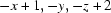.

**Fig. 1.**
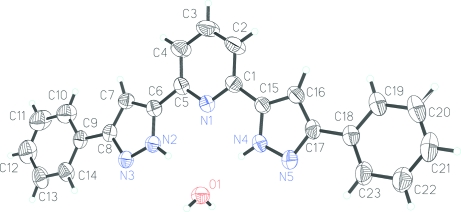
The molecular structure of the title compound, showing the atom-numbering scheme. Displacement ellipsoids are drawn at the 40% probability level. H atoms are shown as small spheres of arbitrary radii.

**Fig. 2.**
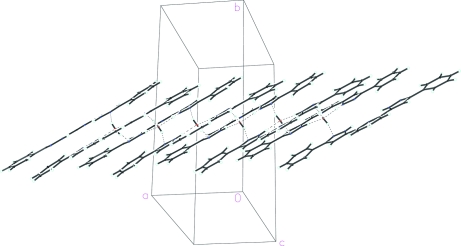
A partial packing view, showing the hydrogen bonds (dashed lines).

## supplementary crystallographic information

### Comment

Pyrazolyl ligands are a kind of mutifuntional organic ligands often displaying *exo*-bidentate coordination mode (Dias & Gamage, 2007; Zhou & Chen, 2007*a*; Zhang *et al.*, 2007). The title compound, 2,6-bis(3-phenyl-1*H*-pyrazol-5-yl)pyridine (hereinafter abbreviated to bppp), is a pincer-like bispyrazolyl ligand. As a kind of anionic multidentate linker, it is a suitable candidate to connect transition metals into aggregate (Zhou & Chen, 2007*b*).

As shown in Fig. 1, the asymmetric unit contains one bppp molecule and one water molecule, and they are linked together *via* N—H···O hydrogen bonds. The pyrazole rings are slightly twisted from the central pyridine ring with the dihedral angles of 5.3 (2) and 3.5 (2)°. The pyrazole and phenyl rings on each side of the molecule are also approximately coplanar with the dihedral angles of 6.0 (2) and 4.5 (2)°. Furthermore, O—H···N hydrogen bonds extend it in a one-dimensional chains running parallel to the *a* axis (Fig. 2). All bond lengths and angles in the title compound are normal.

### Experimental

All reagents were of analytical grade and used without further purification. Bppp was prepared by the general procedure of Zhou and Chen (2007*a*). Bppp (36 mg, 0.1 mmol) in 20 ml of water in a sealed stainless vial was heated at 170 °C for 48 h. Cooling the vial slowly (1 °C/h) afforded colorless crystals. Analysis found: C 72.64, H 4.90, N 18.25%; calculated for C_23_H_19_N_5_O: C 72.42, H 5.02, N 18.36%.

### Refinement

Water H atoms were located in a difference Fourier map and refined isotropically with bond and distance restraints of O—H = 0.83 (1) and H···H = 1.45 (1) Å. Other H atoms were positioned geometrically and treated as riding, with C—H = 0.93 and N—H = 0.86 Å, and with *U*_iso_(H) = 1.2*U*_eq_(C,*N*).

### Crystal data

**Table:**

C_23_H_17_N_5_·H_2_O	*F*_000_ = 800
*M**_r_* = 381.43	*D*_x_ = 1.277 Mg m^−^^3^
Monoclinic, *P*2_1_/*n*	Mo *K*α radiation λ = 0.71073 Å
Hall symbol: -P 2yn	Cell parameters from 1722 reflections
*a* = 8.0581 (17) Å	θ = 2.8–22.2º
*b* = 19.975 (2) Å	µ = 0.08 mm^−^^1^
*c* = 12.451 (2) Å	*T* = 298 (2) K
β = 98.273 (3)º	Needle, colorless
*V* = 1983.2 (6) Å^3^	0.42 × 0.11 × 0.07 mm
*Z* = 4	

### Data collection

**Table:**

Bruker SMART CCD area-detector diffractometer	3513 independent reflections
Radiation source: fine-focus sealed tube	1796 reflections with *I* > 2σ(*I*)
Monochromator: graphite	*R*_int_ = 0.077
*T* = 298(2) K	θ_max_ = 25.1º
φ and ω scans	θ_min_ = 1.9º
Absorption correction: multi-scan(*SADABS*; Sheldrick, 2002)	*h* = −8→9
*T*_min_ = 0.966, *T*_max_ = 0.994	*k* = −22→23
9869 measured reflections	*l* = −14→13

### Refinement

**Table:**

Refinement on *F*^2^	Secondary atom site location: difference Fourier map
Least-squares matrix: full	Hydrogen site location: inferred from neighbouring sites
*R*[*F*^2^ > 2σ(*F*^2^)] = 0.063	H atoms treated by a mixture of independent and constrained refinement
*wR*(*F*^2^) = 0.159	*w* = 1/[σ^2^(*F*_o_^2^) + (0.0411*P*)^2^] where *P* = (*F*_o_^2^ + 2*F*_c_^2^)/3
*S* = 1.05	(Δ/σ)_max_ < 0.001
3513 reflections	Δρ_max_ = 0.18 e Å^−^^3^
271 parameters	Δρ_min_ = −0.18 e Å^−^^3^
3 restraints	Extinction correction: SHELXTL (Sheldrick, 2008), Fc^*^=kFc[1+0.001xFc^2^λ^3^/sin(2θ)]^-1/4^
Primary atom site location: structure-invariant direct methods	Extinction coefficient: 0.0038 (10)

### Special details

**Table:**

Geometry. All e.s.d.'s (except the e.s.d. in the dihedral angle between two l.s. planes) are estimated using the full covariance matrix. The cell e.s.d.'s are taken into account individually in the estimation of e.s.d.'s in distances, angles and torsion angles; correlations between e.s.d.'s in cell parameters are only used when they are defined by crystal symmetry. An approximate (isotropic) treatment of cell e.s.d.'s is used for estimating e.s.d.'s involving l.s. planes.
Refinement. Refinement of *F*^2^ against ALL reflections. The weighted *R*-factor *wR* and goodness of fit *S* are based on *F*^2^, conventional *R*-factors *R* are based on *F*, with *F* set to zero for negative *F*^2^. The threshold expression of *F*^2^ > σ(*F*^2^) is used only for calculating *R*-factors(gt) *etc*. and is not relevant to the choice of reflections for refinement. *R*-factors based on *F*^2^ are statistically about twice as large as those based on *F*, and *R*- factors based on ALL data will be even larger.

### Fractional atomic coordinates and isotropic or equivalent isotropic displacement parameters (Å^2^)

**Table:**

	*x*	*y*	*z*	*U*_iso_*/*U*_eq_	
N1	0.1701 (3)	0.11239 (13)	0.7473 (2)	0.0464 (7)	
N2	0.4662 (3)	0.11019 (14)	0.8827 (2)	0.0509 (7)	
H17	0.3906	0.0825	0.8969	0.061*	
N3	0.6177 (3)	0.11540 (14)	0.9425 (2)	0.0510 (7)	
N4	−0.0667 (3)	0.02481 (13)	0.7985 (2)	0.0489 (7)	
H5	0.0272	0.0252	0.8413	0.059*	
N5	−0.1970 (3)	−0.01481 (14)	0.8133 (2)	0.0535 (8)	
C1	0.0274 (4)	0.10897 (16)	0.6795 (3)	0.0469 (8)	
C2	−0.0007 (5)	0.14642 (18)	0.5851 (3)	0.0671 (11)	
H2	−0.1022	0.1436	0.5392	0.081*	
C3	0.1241 (5)	0.1876 (2)	0.5611 (3)	0.0800 (13)	
H3	0.1081	0.2132	0.4981	0.096*	
C4	0.2718 (5)	0.19125 (18)	0.6292 (3)	0.0705 (11)	
H4	0.3580	0.2188	0.6134	0.085*	
C5	0.2900 (4)	0.15272 (16)	0.7226 (3)	0.0485 (9)	
C6	0.4445 (4)	0.15273 (16)	0.7981 (3)	0.0464 (8)	
C7	0.5920 (4)	0.18745 (16)	0.8030 (3)	0.0491 (9)	
H7	0.6173	0.2208	0.7558	0.059*	
C8	0.6966 (4)	0.16260 (15)	0.8935 (3)	0.0459 (8)	
C9	0.8667 (4)	0.18274 (17)	0.9361 (3)	0.0479 (9)	
C10	0.9428 (5)	0.2353 (2)	0.8903 (3)	0.0759 (12)	
H10	0.8857	0.2576	0.8306	0.091*	
C11	1.1031 (6)	0.2547 (2)	0.9324 (4)	0.0936 (15)	
H11	1.1533	0.2903	0.9013	0.112*	
C12	1.1886 (5)	0.2221 (2)	1.0195 (4)	0.0860 (14)	
H12	1.2972	0.2350	1.0472	0.103*	
C13	1.1139 (5)	0.17056 (19)	1.0656 (4)	0.0744 (12)	
H13	1.1704	0.1492	1.1265	0.089*	
C14	0.9565 (4)	0.15005 (17)	1.0232 (3)	0.0607 (10)	
H14	0.9091	0.1134	1.0535	0.073*	
C15	−0.0992 (4)	0.06365 (16)	0.7095 (2)	0.0454 (8)	
C16	−0.2579 (4)	0.04886 (16)	0.6641 (3)	0.0506 (9)	
H16	−0.3166	0.0673	0.6013	0.061*	
C17	−0.3159 (4)	0.00052 (17)	0.7303 (3)	0.0474 (8)	
C18	−0.4788 (4)	−0.03241 (18)	0.7192 (3)	0.0510 (9)	
C19	−0.6077 (4)	−0.01190 (19)	0.6401 (3)	0.0617 (10)	
H19	−0.5885	0.0225	0.5930	0.074*	
C20	−0.7635 (5)	−0.0415 (2)	0.6298 (3)	0.0717 (12)	
H20	−0.8480	−0.0271	0.5759	0.086*	
C21	−0.7945 (5)	−0.0916 (2)	0.6984 (4)	0.0772 (13)	
H21	−0.9002	−0.1112	0.6919	0.093*	
C22	−0.6695 (5)	−0.1130 (2)	0.7767 (3)	0.0756 (12)	
H22A	−0.6894	−0.1480	0.8225	0.091*	
C23	−0.5147 (5)	−0.0831 (2)	0.7880 (3)	0.0668 (11)	
H23A	−0.4319	−0.0973	0.8432	0.080*	
O1	0.2448 (3)	0.01202 (14)	0.9621 (2)	0.0566 (7)	
H22	0.216 (6)	0.028 (2)	1.019 (2)	0.15 (2)*	
H23	0.298 (6)	−0.0242 (14)	0.967 (3)	0.15 (2)*	

### Atomic displacement parameters (Å^2^)

**Table:**

	*U*^11^	*U*^22^	*U*^33^	*U*^12^	*U*^13^	*U*^23^
N1	0.0427 (17)	0.0464 (17)	0.0497 (16)	0.0026 (14)	0.0054 (14)	−0.0008 (13)
N2	0.0401 (18)	0.0554 (18)	0.0563 (17)	−0.0080 (14)	0.0035 (14)	0.0059 (15)
N3	0.0378 (17)	0.0568 (18)	0.0577 (17)	−0.0084 (14)	0.0040 (14)	0.0026 (14)
N4	0.0354 (17)	0.0575 (18)	0.0516 (17)	−0.0011 (14)	−0.0011 (13)	0.0039 (14)
N5	0.0397 (17)	0.0625 (19)	0.0577 (18)	−0.0063 (15)	0.0048 (15)	0.0025 (15)
C1	0.050 (2)	0.041 (2)	0.049 (2)	0.0068 (17)	0.0032 (18)	−0.0019 (16)
C2	0.063 (3)	0.059 (2)	0.071 (3)	−0.005 (2)	−0.017 (2)	0.020 (2)
C3	0.084 (3)	0.076 (3)	0.074 (3)	−0.014 (3)	−0.011 (3)	0.035 (2)
C4	0.063 (3)	0.067 (3)	0.077 (3)	−0.016 (2)	−0.006 (2)	0.026 (2)
C5	0.042 (2)	0.044 (2)	0.058 (2)	0.0012 (17)	0.0023 (18)	−0.0016 (17)
C6	0.046 (2)	0.0412 (19)	0.052 (2)	0.0007 (17)	0.0053 (17)	−0.0032 (17)
C7	0.052 (2)	0.0403 (19)	0.056 (2)	−0.0025 (17)	0.0114 (18)	0.0037 (17)
C8	0.043 (2)	0.039 (2)	0.057 (2)	−0.0035 (16)	0.0130 (18)	−0.0073 (16)
C9	0.041 (2)	0.044 (2)	0.060 (2)	−0.0072 (16)	0.0103 (18)	−0.0101 (17)
C10	0.071 (3)	0.077 (3)	0.078 (3)	−0.026 (2)	0.004 (2)	0.004 (2)
C11	0.085 (4)	0.098 (4)	0.097 (3)	−0.046 (3)	0.010 (3)	0.010 (3)
C12	0.056 (3)	0.080 (3)	0.121 (4)	−0.028 (2)	0.009 (3)	−0.009 (3)
C13	0.049 (2)	0.065 (3)	0.107 (3)	−0.005 (2)	0.001 (2)	−0.006 (2)
C14	0.044 (2)	0.048 (2)	0.087 (3)	−0.0069 (18)	0.002 (2)	0.002 (2)
C15	0.042 (2)	0.050 (2)	0.0436 (19)	0.0046 (17)	0.0033 (16)	−0.0001 (17)
C16	0.045 (2)	0.057 (2)	0.048 (2)	0.0066 (18)	−0.0010 (17)	0.0009 (18)
C17	0.038 (2)	0.055 (2)	0.049 (2)	0.0046 (17)	0.0073 (17)	−0.0090 (17)
C18	0.044 (2)	0.058 (2)	0.050 (2)	0.0012 (18)	0.0064 (17)	−0.0136 (18)
C19	0.044 (2)	0.071 (3)	0.068 (3)	0.004 (2)	0.001 (2)	−0.015 (2)
C20	0.045 (3)	0.086 (3)	0.080 (3)	0.008 (2)	−0.006 (2)	−0.030 (3)
C21	0.048 (3)	0.101 (4)	0.085 (3)	−0.015 (2)	0.019 (2)	−0.034 (3)
C22	0.058 (3)	0.096 (3)	0.073 (3)	−0.013 (2)	0.011 (2)	−0.007 (2)
C23	0.043 (2)	0.087 (3)	0.070 (3)	−0.012 (2)	0.006 (2)	−0.004 (2)
O1	0.0573 (17)	0.0555 (17)	0.0564 (16)	−0.0011 (14)	0.0067 (13)	0.0061 (13)

### Geometric parameters (Å, °)

**Table:**

N1—C5	1.327 (4)	C10—H10	0.9300
N1—C1	1.327 (4)	C11—C12	1.364 (6)
N2—N3	1.339 (3)	C11—H11	0.9300
N2—C6	1.344 (4)	C12—C13	1.360 (5)
N2—H17	0.8600	C12—H12	0.9300
N3—C8	1.333 (4)	C13—C14	1.365 (5)
N4—C15	1.348 (4)	C13—H13	0.9300
N4—N5	1.348 (3)	C14—H14	0.9300
N4—H5	0.8600	C15—C16	1.354 (4)
N5—C17	1.340 (4)	C16—C17	1.393 (4)
C1—C2	1.383 (4)	C16—H16	0.9300
C1—C15	1.453 (4)	C17—C18	1.457 (4)
C2—C3	1.365 (5)	C18—C23	1.383 (5)
C2—H2	0.9300	C18—C19	1.387 (4)
C3—C4	1.360 (5)	C19—C20	1.376 (5)
C3—H3	0.9300	C19—H19	0.9300
C4—C5	1.385 (4)	C20—C21	1.362 (5)
C4—H4	0.9300	C20—H20	0.9300
C5—C6	1.448 (4)	C21—C22	1.366 (5)
C6—C7	1.370 (4)	C21—H21	0.9300
C7—C8	1.397 (4)	C22—C23	1.372 (5)
C7—H7	0.9300	C22—H22A	0.9300
C8—C9	1.454 (4)	C23—H23A	0.9300
C9—C14	1.378 (4)	O1—H22	0.84 (3)
C9—C10	1.380 (5)	O1—H23	0.84 (4)
C10—C11	1.378 (5)		
			
C5—N1—C1	118.5 (3)	C12—C11—H11	119.8
N3—N2—C6	113.1 (3)	C10—C11—H11	119.8
N3—N2—H17	123.5	C13—C12—C11	119.6 (4)
C6—N2—H17	123.5	C13—C12—H12	120.2
C8—N3—N2	104.9 (3)	C11—C12—H12	120.2
C15—N4—N5	112.7 (3)	C12—C13—C14	120.4 (4)
C15—N4—H5	123.6	C12—C13—H13	119.8
N5—N4—H5	123.6	C14—C13—H13	119.8
C17—N5—N4	104.2 (3)	C13—C14—C9	121.1 (4)
N1—C1—C2	122.2 (3)	C13—C14—H14	119.5
N1—C1—C15	116.4 (3)	C9—C14—H14	119.5
C2—C1—C15	121.3 (3)	N4—C15—C16	106.1 (3)
C3—C2—C1	118.4 (4)	N4—C15—C1	120.6 (3)
C3—C2—H2	120.8	C16—C15—C1	133.2 (3)
C1—C2—H2	120.8	C15—C16—C17	106.4 (3)
C4—C3—C2	120.2 (4)	C15—C16—H16	126.8
C4—C3—H3	119.9	C17—C16—H16	126.8
C2—C3—H3	119.9	N5—C17—C16	110.5 (3)
C3—C4—C5	118.1 (4)	N5—C17—C18	120.3 (3)
C3—C4—H4	120.9	C16—C17—C18	129.2 (3)
C5—C4—H4	120.9	C23—C18—C19	117.0 (3)
N1—C5—C4	122.6 (3)	C23—C18—C17	122.5 (3)
N1—C5—C6	115.9 (3)	C19—C18—C17	120.4 (3)
C4—C5—C6	121.5 (3)	C20—C19—C18	121.2 (4)
N2—C6—C7	105.7 (3)	C20—C19—H19	119.4
N2—C6—C5	120.7 (3)	C18—C19—H19	119.4
C7—C6—C5	133.5 (3)	C21—C20—C19	120.3 (4)
C6—C7—C8	106.0 (3)	C21—C20—H20	119.8
C6—C7—H7	127.0	C19—C20—H20	119.8
C8—C7—H7	127.0	C20—C21—C22	119.7 (4)
N3—C8—C7	110.3 (3)	C20—C21—H21	120.2
N3—C8—C9	121.1 (3)	C22—C21—H21	120.2
C7—C8—C9	128.6 (3)	C21—C22—C23	120.2 (4)
C14—C9—C10	118.1 (3)	C21—C22—H22A	119.9
C14—C9—C8	121.1 (3)	C23—C22—H22A	119.9
C10—C9—C8	120.8 (3)	C22—C23—C18	121.5 (4)
C11—C10—C9	120.3 (4)	C22—C23—H23A	119.2
C11—C10—H10	119.8	C18—C23—H23A	119.2
C9—C10—H10	119.8	H22—O1—H23	118 (2)
C12—C11—C10	120.4 (4)		
			
C6—N2—N3—C8	−0.7 (4)	C9—C10—C11—C12	0.3 (7)
C15—N4—N5—C17	−0.5 (3)	C10—C11—C12—C13	−0.8 (7)
C5—N1—C1—C2	−0.7 (5)	C11—C12—C13—C14	2.1 (7)
C5—N1—C1—C15	179.4 (3)	C12—C13—C14—C9	−3.0 (6)
N1—C1—C2—C3	0.7 (6)	C10—C9—C14—C13	2.5 (5)
C15—C1—C2—C3	−179.4 (3)	C8—C9—C14—C13	−177.6 (3)
C1—C2—C3—C4	−0.1 (6)	N5—N4—C15—C16	0.1 (4)
C2—C3—C4—C5	−0.5 (6)	N5—N4—C15—C1	−179.1 (3)
C1—N1—C5—C4	0.1 (5)	N1—C1—C15—N4	−3.8 (4)
C1—N1—C5—C6	−178.2 (3)	C2—C1—C15—N4	176.4 (3)
C3—C4—C5—N1	0.6 (6)	N1—C1—C15—C16	177.3 (3)
C3—C4—C5—C6	178.7 (3)	C2—C1—C15—C16	−2.6 (6)
N3—N2—C6—C7	0.4 (4)	N4—C15—C16—C17	0.3 (4)
N3—N2—C6—C5	178.1 (3)	C1—C15—C16—C17	179.3 (3)
N1—C5—C6—N2	4.9 (4)	N4—N5—C17—C16	0.7 (3)
C4—C5—C6—N2	−173.4 (3)	N4—N5—C17—C18	−179.5 (3)
N1—C5—C6—C7	−178.1 (3)	C15—C16—C17—N5	−0.6 (4)
C4—C5—C6—C7	3.6 (6)	C15—C16—C17—C18	179.5 (3)
N2—C6—C7—C8	0.0 (3)	N5—C17—C18—C23	−4.3 (5)
C5—C6—C7—C8	−177.2 (3)	C16—C17—C18—C23	175.6 (3)
N2—N3—C8—C7	0.7 (4)	N5—C17—C18—C19	173.4 (3)
N2—N3—C8—C9	179.6 (3)	C16—C17—C18—C19	−6.7 (5)
C6—C7—C8—N3	−0.5 (4)	C23—C18—C19—C20	−0.8 (5)
C6—C7—C8—C9	−179.2 (3)	C17—C18—C19—C20	−178.6 (3)
N3—C8—C9—C14	5.3 (5)	C18—C19—C20—C21	0.4 (6)
C7—C8—C9—C14	−176.1 (3)	C19—C20—C21—C22	−0.8 (6)
N3—C8—C9—C10	−174.8 (3)	C20—C21—C22—C23	1.6 (6)
C7—C8—C9—C10	3.9 (5)	C21—C22—C23—C18	−2.1 (6)
C14—C9—C10—C11	−1.2 (6)	C19—C18—C23—C22	1.6 (5)
C8—C9—C10—C11	178.9 (4)	C17—C18—C23—C22	179.4 (3)

### Hydrogen-bond geometry (Å, °)

**Table:**

*D*—H···*A*	*D*—H	H···*A*	*D*···*A*	*D*—H···*A*
O1—H22···N5^i^	0.84 (3)	2.13 (3)	2.878 (4)	148 (4)
O1—H23···N3^ii^	0.84 (4)	2.20 (2)	2.956 (4)	150 (4)
N2—H17···O1	0.86	2.07	2.918 (4)	167
N4—H5···O1	0.86	2.16	3.007 (4)	170

Symmetry codes: (i) −*x*, −*y*, −*z*+2; (ii) −*x*+1, −*y*, −*z*+2.

## References

[bb1] Bruker (2001). *SMART* and *SAINT* Bruker AXS Inc., Madison, Wisconsin, USA.

[bb2] Dias, H. V. R. & Gamage, C. S. P. (2007). *Angew. Chem. Int. Ed.* **46**, 2192–2194. 10.1002/anie.200604585 17286331

[bb3] Sheldrick, G. M. (2002). *SADABS* University of Göttingen, Germany.

[bb4] Sheldrick, G. M. (2008). *Acta Cryst.* A**64**, 112–122. 10.1107/S0108767307043930 18156677

[bb5] Westrip, S. P. (2008). *publCIF.* In preparation.

[bb6] Zhang, J.-P., Horike, S. & Kitagawa, S. (2007). *Angew. Chem. Int. Ed.* **46**, 889–892. 10.1002/anie.200603270 17183498

[bb7] Zhou, Y. B. & Chen, W. Z. (2007*a*). *Dalton Trans. *pp*.* 5123-5125. 10.1039/b712411b 17985018

[bb8] Zhou, Y. B. & Chen, W. Z. (2007*b*). *Organometallics*, **26**, 2742–2746.

